# Trajectories of Adherence to Study-Prescribed Physical Activity Goals in a mHealth Weight Loss Intervention

**DOI:** 10.3390/s25247595

**Published:** 2025-12-15

**Authors:** Zhadyra Bizhanova, Lora E. Burke, Maria M. Brooks, Bonny Rockette-Wagner, Jacob K. Kariuki, Susan M. Sereika

**Affiliations:** 1 School of Nursing, University of Pittsburgh, 3500 Victoria Building, Pittsburgh, PA 15261, USA; 2 School of Public Health, University of Pittsburgh, 130 De Soto Street, Pittsburgh, PA 15261, USA; mbrooks@pitt.edu (M.M.B.);; 3 Nell Hodgson Woodruff School of Nursing, Emory University, 1520 Clifton Road NE, Atlanta, GA 30322, USA

**Keywords:** physical activity guidelines, mobile health, wearable physical activity trackers, group-based trajectory modeling, predictors, adherence, weight loss, obesity

## Abstract

**Highlights:**

**What are the main findings?**

- Four distinct physical activity goal-adherence trajectories were identified in adults with overweight/obesity.
- Higher physical activity goal adherence was associated with greater weight loss.

**What are the implications of the main findings?**

- Early monitoring of physical activity adherence can identify individuals at risk of not meeting behavioral goals and allow timely, targeted support.
- Older adults and men may respond more positively to remotely delivered mHealth interventions using wearable activity trackers.

**Abstract:**

**Introduction**: Engaging in ≥300 min/week of moderate-to-vigorous physical activity (MVPA) is recommended for weight management. This study identified MVPA goal-adherence trajectories and associated predictors and weight outcomes in a 12-month mHealth weight-loss trial. **Materials and Methods**: This was a secondary analysis of valid PA data (≥4 days/week with ≥500 steps/day) from participants (age ≥ 18 years, BMI 27–43 kg/m^2^) randomized 1:1 to self-monitoring with tailored feedback or self-monitoring only. Both groups received Fitbit trackers. Group-based trajectory modeling identified adherence trajectories and baseline predictors. Analysis of variance was used to estimate associations between trajectory group membership and 12-month weight change. **Results**: Among 502 participants (79% female, 82% White, mean age of 45.0 ±14.4 years), four MVPA goal-adherence trajectories were identified: lower stable (34.5%), moderate (39.8%), increasing (19.3%), and high (6.4%). A graded association was observed with better adherence trajectories being associated with greater 12-month weight loss (*p* < 0.0001). Older age, male sex, being unpartnered, and higher first-week MVPA predicted membership in higher adherence trajectory groups (*p* < 0.05). **Conclusions**: Higher MVPA goal-adherence was related to greater weight loss. Early MVPA levels predicted long-term adherence, supporting the importance of personalized, technology-supported strategies to promote long-term PA adherence and inform targeted interventions to prevent chronic diseases.

## 1. Introduction

In 2022, 43% of adults globally were overweight, and 16% had obesity [1]. In the US, obesity prevalence was 39% in men and 41% in women in 2024, with severe obesity prevalence higher in women (12%) compared to men (7%) [2]. Obesity is a major risk factor for cardiovascular disease, type 2 diabetes, and several types of cancers [3,4,5]. The aim of behavioral weight loss interventions is to prevent further weight gain or achieve weight loss to reduce the risk of developing these chronic disorders [5]. Behavioral weight loss interventions typically combine caloric restriction with increased physical activity (PA), particularly aerobic or combined aerobic-resistance exercises, with moderate effectiveness in treating obesity [6]. The key strategy in behavioral weight-loss interventions is self-monitoring of PA, body weight, and food intake, supported by wearable activity trackers, digital weight scales, and smartphone apps, respectively [7]. Participant engagement in tracking and reviewing behaviors in relation to personal health goals can promote self-regulation of healthy lifestyle behaviors.

Aerobic PA is commonly used in weight loss interventions. When performed at higher levels and combined with dietary changes, it can support weight loss and maintenance [8]. Guidelines recommend ≥150 min/week of moderate-to-vigorous PA (MVPA) for modest weight loss and 150–300 min/week for greater benefits [9]. A random effects analysis of 116 randomized controlled trials found that ≥150 min/week of MVPA reduced weight (−3.47 kg), waist circumference (−2.06 cm), and body fat (−2.51 kg), with even greater reductions at ≥300 min/week [10]. However, in 2020, only 22.7% of US adults met the aerobic guideline [11].

Wearable activity trackers offer a low-burden way to support long-term adherence to MVPA goals [12]. However, sustaining engagement with device-supported interventions remains a challenge due to issues such as device maintenance, synchronization, and use of multiple trackers [13]. A 12-month study showed significant declines in Fitbit use and PA adherence after six months, underscoring the need for strategies to sustain engagement [14].

Although mHealth interventions have demonstrated short-term improvements in PA and weight outcomes, most prior studies have been limited to durations of less than six months and have focused primarily on immediate changes (e.g., steps, MVPA, and weight) [15,16,17]. This leaves a critical gap in understanding long-term patterns of MVPA goal adherence and their relationship to weight loss. Identifying distinct adherence trajectories can inform the development of personalized digital strategies that target individuals at risk of low and declining PA and support sustained behavior change. Group-based trajectory modeling (GBTM) addresses this need by identifying distinct longitudinal trajectories and capturing heterogeneity in response to interventions, rather than relying on single measurements [18]. While prior work, such as Imes et al. [19] and Adachi et al. [20], has applied GBTM to step-count trajectories, limited evidence remains on MVPA goal-adherence trajectories from large, longitudinal datasets using commercial wearables among middle- and older-aged adults with overweight or obesity in remotely delivered mHealth interventions. The current secondary analysis addresses this gap by identifying MVPA goal-adherence trajectories over 12 months within the SMARTER trial, a 12-month randomized controlled trial (RCT) comparing self-monitoring (SM) alone to SM with tailored feedback (SM + FB) on diet, PA, and weight.

The current study had three aims: (1) identify distinct trajectories of adherence to the study-prescribed MVPA goal of ≥300 min/week over 12 months, (2) determine whether baseline characteristics and first-week MVPA predicted trajectory group membership, and (3) assess whether trajectory group membership was associated with percent weight change at 12 months. Based on prior evidence, we hypothesized a priori that there would be three [20] to four [19] distinct adherence trajectories, as previous step-based behavioral interventions using GBTM identified a small number of discrete temporal patterns. We also hypothesized that higher MVPA goal-adherence trajectories would be associated with greater weight loss [21], older age [20], male sex [15,22], being single [23], and higher initial MVPA [24,25]. Findings may inform personalized mHealth interventions to improve long-term adherence to MVPA goals and reduce the risks of obesity-related chronic diseases.

## 2. Methods

Study Design: SMARTER was a two-arm, parallel-group RCT conducted from August 2018 to March 2021 to evaluate the efficacy of two behavioral interventions on weight loss over 12 months [26,27]. Participants were randomized to either self-monitoring plus feedback (SM + FB) or self-monitoring only (SM). Both groups used Fitbit Charge 2™ trackers (San Francisco, CA, USA), the Fitbit app for dietary tracking, and study-issued (Withings, Issy-les-Moulineaux, France) home scales. The SMARTER app delivered tailored feedback messages three times daily for 12 months, using a fixed rule-based algorithm that selected the most relevant message from a monthly updated library based on real-time self-monitoring data (i.e., PA, diet, and weight) [27]. Messages focused on one behavior at a time, with up to three daily diet messages, three weekly PA messages, and one weekly weight message.

Participants were instructed to achieve ≥150 min/week of MVPA during the first 12 weeks, gradually increasing by 10 min/week to reach ≥300 min/week by week 42, and maintained through week 52 [26]. Mostly walking, and other aerobic activities, such as bicycling and jogging, were encouraged. All participants received Fitbit motivational features (badges, feedback, summaries), but only participants in the SM + FB received tailored feedback.

Sample: The sample comprised 502 participants recruited from southwestern Pennsylvania between August 2018 and March 2020 using the Pitt + Me registry, university email announcements, electronic and postal mailings, social media posts, community flyers, and collaboration with primary care practices [26]. Eligibility criteria included body mass index (BMI) 27–43 kg/m^2^, completion of a five-day electronic dietary diary, smartphone ownership with a data plan, and ability to perform moderate-intensity PA. Exclusion criteria included need for supervised interventions, pregnancy plans, serious mental illness or eating disorder, high alcohol intake, or participation in another weight loss program. Of 1741 individuals screened, 1150 were excluded for the following reasons: BMI outside range (n = 214), age < 18 years (n = 3), incomplete food diary (n = 214), discontinued screening (n = 431), heavy drinking (n = 4), breastfeeding (n = 13), history of bariatric surgery (n = 39), using a weight loss app (n = 26) or participation in a weight loss program (n = 61), general health history (n = 51), Eating Habits Checklist score ≥ 37 (n = 5), not having a smartphone (n = 7), and change in antidepressant use in past 6 months (n = 82) [27]. After baseline assessment (n = 591), an additional 89 participants were excluded due to BMI outside the range (n = 10) and discontinued baseline (n = 79), resulting in 502 randomized participants. The full CONSORT flowchart detailing screening, exclusions, and randomization has been published elsewhere [27]. All participants provided verbal and written informed consent and were instructed to complete in-person assessments at baseline, 6 months, and 12 months. The study was approved by the Institutional Review Board of the University of Pittsburgh (IRB is #19060112, protocol code is PRO17040453 from April 2017, NIH funding: R01-HL131583, ClinicalTrials.gov: NCT03367936), and the study design and methodology have been published previously [26].

Procedures: At baseline, participants received in-person assessments and 1:1 behavioral counseling on SM and goal setting [26]. Calorie goals were based on sex and weight (women: 1200 or 1500 kcal/day; men: 1500 or 1800 kcal/day) and adjusted as needed. Participants tracked diet, PA, and weight daily. Fitbit trackers were used for PA due to their accuracy and practicality in free-living conditions. Adherence to PA self-monitoring or Fitbit wear was defined as logging ≥500 steps/day. This threshold was selected to confirm device wear rather than activity, as it reflects minimal movement and distinguishes non-wear from sedentary behavior. This criterion has been used in prior Fitbit-supported interventions [28]. Adherence to the weight self-monitoring was defined as recording a daily weight, while adherence to the dietary self-monitoring was defined as logging ≥50% of the daily calorie goal in the Fitbit app. This threshold was based on prior research showing that meeting at least half of the prescribed calorie goal was significantly associated with weight loss over 12 months [26,27].

SM data were uploaded to the study-developed Awesome Data Acquisition Method (ADAM), which employed automated monitoring to ensure data quality [29]. For example, alerts were generated if no diet, weight, or PA were recorded for seven consecutive days or if a participant’s weight changed by more than five pounds within a week, prompting the study team to identify and address any potential device or synchronization issue or the correctness of a significant change in weight. For SM + FB participants, the study-developed SMARTER app algorithm issued up to three feedback messages per day. Particularly, the SMARTER app algorithm randomly delivered prompts to participants’ smartphones during morning, afternoon, and evening hours. If a participant opened the prompt within one hour, the app displayed a personalized FB message based on their tracked data, which was recorded as an “opened message” [30]. If the prompt was not opened within that time, no feedback was shown, and it was recorded as a “missed message.”

Measurements: The primary outcome was the percentage of adherence to the study-prescribed MVPA goal of ≥300 min/week, calculated as (Weekly MVPA minutes/300 min/week) × 100%.

MVPA was computed from Fitbit data by summing minutes classified as “fairly active” and “very active,” which correspond to estimated energy expenditures of 3 to 6 METs and greater than 6 METs, respectively [31]. These thresholds align with standard definitions of MVPA [32]. Weeks were considered valid for PA measurement if they included ≥4 wear days, consistent with commonly accepted criteria for valid PA data in adults [33,34]. Weeks that did not meet the PA criterion were deemed invalid and excluded from adherence calculations. Weekly MVPA was estimated by aggregating MVPA, dividing by valid days, and multiplying by seven to account for potential underestimation of typical adherence. We conducted an additional analysis using group-based trajectory modeling based on the average percentage of MVPA goal adherence. This measure was calculated as weekly MVPA divided by the number of valid wear days, without extrapolation to seven days of 300 min/week, and multiplied by 100%. The results of this analysis are presented in Supplemental File S1.

The MVPA goal of at least 300 min/week was selected as the primary outcome threshold because it aligns with the upper end of the 2018 U.S. Physical Activity Guideline, which recommends 150 to 300 min per week for substantial health benefits, particularly for weight loss [9]. Since participants were instructed to progressively increase MVPA from 150 min/week during the initial 12 weeks to 300 min/week by week 42, we used the 300 min threshold to evaluate long-term adherence. This approach allowed uniform evaluation of MVPA adherence trajectories, as each participant began the study at different initial MVPA levels and followed individual trajectories toward the ultimate MVPA goal.

The secondary outcome of percent weight change was calculated as ([Weight at 12 month − weight at baseline]/weight at baseline) × 100%.

Weight was measured in kilograms using a Tanita scale (Model BF-350, Tanita Corporation, Tokyo, Japan) at baseline and at 6 and 12 months for in-person assessment visits. All participants had in-person baseline weight data measured on the Tanita research scale. Following the COVID-19 pandemic, weight assessments were performed remotely using the study-issued home scale, calibrated against the research scale before distribution to the participant. As for the remote assessments, participants were instructed to weigh themselves at home, wearing light clothing and no shoes.

Predictors included baseline age, sex, partner status, baseline BMI, first-week MVPA (used as a proxy for baseline PA of one’s PA prior to randomization), and timing of follow-up relative to COVID-19 lockdown (binary variable if assessments occurred after 17 March 2020). Predictor selection was based on prior literature [15,20,21,23,24,25] and preliminary analyses using machine learning and regression approaches (i.e., random forest (RF), regression trees, and LASSO) to construct prediction models for PA adherence, considering 25 baseline characteristics and short-term response predictors to reduce overfitting [30]. Intervention fidelity was measured by the number of FB messages opened. Engagement with PA feedback for SM + FB was calculated as (Number of PA messages opened over 12 months/Number of PA messages scheduled to be sent over 12 months) × 100%.

Statistical Analysis: All analyses were conducted using SAS 9.4 (SAS Institute, Inc., Cary, NC, USA). Baseline characteristics, first-week MVPA, and 12-month PA engagement were summarized as mean ± SD or median (Q1–Q3) for continuous variables and frequencies (%) for categorical variables. Since MVPA levels had right-skewed distributions, we report both mean (±SD) and median (Q1, Q3) for the first week in Table 1 and applied a square-root transformation in the final model.

Group-based trajectory modeling (GBTM) using PROC TRAJ was used to identify distinct MVPA goal-adherence trajectories. The number of trajectory groups was selected using commonly recommended fit indices that balance model fit and complexity: Bayesian Information Criteria (BIC_1_: the standard BIC calculated from the full model likelihood, BIC_2_: an adjusted BIC that accounts for the number of observations per individual), and Akaike’s Information Criterion (AIC). In addition, practical considerations, such as maintaining a minimum group size of at least 5%, were applied to ensure model adequacy and usefulness [18]. GBTM began with quintic polynomials for each trajectory group and was simplified until the highest-order term remained significant (*p* < 0.05).

Model adequacy was assessed using these diagnostics measures: (1) average posterior probabilities (APP) ≥ 70 for each group, (2) odds of correct classification (OCC) ≥ 5.0 for each group, (3) alignment between estimated group probabilities (π) and observed group proportions, and (4) narrow confidence intervals for estimated group probabilities [18]. The final model estimates, standard errors (SE), adjusted odds ratios (AOR), 95% confidence intervals (CI), π values, t-values, *p*-values, BIC_1_, BIC_2_, AIC, and log-likelihood statistics were reported. The GBTM building process with model fit statistics was described in Supplemental File S1.

Time-invariant predictors included sociodemographic factors (age, race, sex, employment, partner status, and education), health conditions (the Center for Epidemiologic Studies Depression [CES-D] score, history of hypertension, elevated cholesterol, triglycerides, cancer, digestive diseases, mental health problems, autoimmune diseases, osteoarthritis, respiratory diseases, and sleep apnea) [26], exercise-related factors (self-efficacy scores for sticking to exercise and making time for exercise from an 11-item Self-Efficacy and Exercise Habits Survey) [30], behavioral factors (12-month PA messages opened, weekend Fitbit use, follow-up during the COVID-19 pandemic), baseline BMI, and first-week MVPA. Predictor selection was based on the prior literature, preliminary analyses [15,20,21,22,23,24,25], and machine learning and regression approaches (RF, regression trees, and LASSO) [30] and statistical significance (*p* < 0.05). To reduce overfitting, the sample was randomly split 4:1 into training (n = 401) and testing (n = 101) sets. RF, regression trees, and LASSO models were trained on the training set, and predictive performance was evaluated on the testing set using out-of-sample R^2^ for RF and regression trees and R^2^ for LASSO. The final model included treatment group, age, sex, partner status, the COVID-19 indicator, and first-week MVPA (square-root transformed). In the final model, *p*-values were adjusted using FDR (False Discovery Rate) correction to account for multiple testing. Additionally, mean predicted values for adherence to MVPA goals at weeks 1, 26, and 52 by trajectory group membership have been reported in Supplemental File S1.

Percent weight change at 12 months was compared across MVPA goal-adherence trajectory groups using one-way analysis of variance (ANOVA). Statistical significance was evaluated using F-statistics and Type III sums of squares.

Handling of missing data: Missing weight data were imputed using data from study-issued home scales within two weeks of the missed scheduled assessments. All participants had baseline weight data. If data from study-issued home scales were not available, weight was imputed by adding 0.01 kg/day to the last recorded weight, following Wadden’s conservative approach, which assumes a weight regain of 0.3 kg/month after discontinuation of self-monitoring [35]. At 12 months, 38.0% provided in-person data, 41.0% remote data from study-issued home scales, and 21.0% had missing data. The amount and pattern of missingness did not differ significantly between treatment groups. Basic characteristics of SMARTER participants by study completion status are reported in Supplemental File S2. As part of a sensitivity analysis, we excluded participants without both in-person and study-issued home scale weight measurements at 12 months (21%). To assess the significance of weight changes by trajectory group membership at 12 months, we estimated Cohen’s d effect sizes, comparing the lower-stable adherence group with the other three groups.

## 3. Results

The sample (N = 502) was mostly female (79.5%) and White (82.5%) with a mean ± SD age of 45.0 ± 14.4 years and a mean BMI of 33.7 ± 4.0 kg/m^2^ (see Table 1). Most had at least a college degree, with an average of 16.4 ± 2.8 years of education. The median first-week MVPA was 214 min (Q1–Q3: 118–346), and the mean was 244.1 min (SD: 171.9). Almost one third of participants met the MVPA goal of ≥300 min/week during the first week. In the SM + FB group, participants opened, on average, 67.0 ± 32.5% of PA feedback messages over 12 months (Table 1). Of the 502 participants enrolled, 394 (79%) completed the 12-month assessment, resulting in an overall attrition rate of 21%. Participants who dropped out of the study were more likely to be younger, have a higher BMI at baseline, and have lower first-week MVPA levels (all *p*-values < 0.05) (see Supplemental File S2, Table S1).

**Figure 1:**
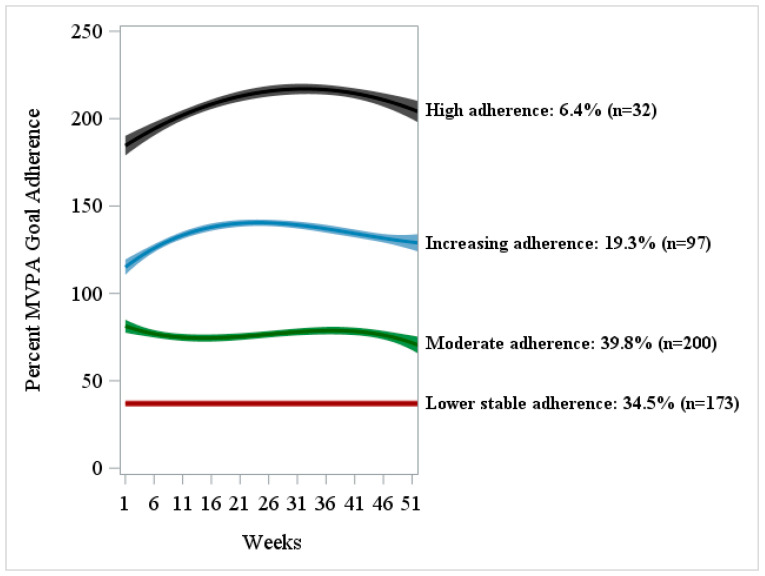
Estimated trajectories of percentage adherence to the study-prescribed MVPA goals with 95% confidence bands over 12 months from the final group-based trajectory modeling with risk factors. *Notes*: MVPA = Moderate-to-Vigorous-Intensity Physical Activity. The percentage of adherence to MVPA goals was computed using the following formula: ((fairly active and very active minutes) × 7 days)/(number of days meeting ≥ 500 daily steps for ≥4 days/week) over ≥300 min/week × 100%.

Four distinct MVPA goal-adherence trajectories were identified over 12 months (see Table 2, Figure 1). The first group (34.5%, n = 173) demonstrated lower and stable adherence, showing no significant change over time (intercept only). The second group (39.8%, n = 200) showed moderate adherence with a curvilinear trajectory (cubic pattern). The third group (19.3%, n = 97) exhibited increasing adherence with curvilinear changes over time (cubic pattern). The fourth group (6.4%, n = 32) maintained consistently high adherence with a quadratic-shaped trajectory over time. Supplemental File S1 (see Table S4–S6, Figure S2) presents group-based trajectory modeling results based on the average percentage of adherence to the MVPA goal, which were consistent with the primary analysis.

Table 3 reports adjusted odds ratios (AORs) for associations between baseline predictors and trajectory group membership, with the lower stable adherence group as the reference. The final GBTM model included treatment group, age, sex, partner status, the COVID-19 pandemic indicator, and first-week MVPA. Older age and higher first-week MVPA were significantly associated with greater odds of belonging to the moderate, increasing, and high adherence groups. Partner status was significantly associated with the increasing group, with partnered individuals having lower odds of belonging to this group. Sex was significantly associated with the rising and high adherence groups, with males having higher odds of belonging to these groups. Participants assessed during the COVID-19 pandemic had lower odds of being in the increasing adherence group; however, the pandemic indicator was not significantly associated with trajectory group membership after FDR adjustment for multiple testing. There was no significant association between randomized group assignment and trajectory group membership (*p*-value ≥ 0.05). The results of GBTM with time-invariant/baseline risk factors, parameter estimates, standard errors, AORs with 95% confidence intervals (CIs), t-values, and *p*-values are reported in Table 3.

For the full sample (N = 502), mean percent weight (95% CI) differed significantly across MVPA trajectory group membership (F = 9.9, *p* < 0.0001; see Table 4). The Lower stable adherence group showed minimal change −0.33% (−1.41, 0.74). Compared to this group, the Moderate adherence group lost −2.59% (−3.58, −1.59; Cohen’s *d* = 0.32), the Increasing adherence group lost −4.29% (−5.73, −2.86; *d* = 0.55), and the High adherence group lost −6.16% (−8.66, −3.66; *d* = 0.71) at 12 months.

Among completers only (n = 394), patterns were similar. The Lower stable adherence group lost −1.87% (−3.12, −0.62). Compared to this group, the Moderate adherence group lost −3.59% (−4.68, −2.49; *d* = 0.26), the Increasing adherence group lost −4.77% (−6.24, −3.30; *d* = 0.44), and the High adherence group lost −6.82% (−9.37, −4.27; *d* = 0.75).

## 4. Discussion

Identifying four distinct MVPA goal-adherence trajectories demonstrates that a “one-size-fits-all” approach does not work in mHealth weight loss programs. Participants followed lower, stable, moderate, increasing, and high adherence patterns over 12 months. Older age and higher initial MVPA predicted membership in higher-adherence trajectory groups, whereas being female or partnered reduced the odds of belonging to the increasing-adherence group. Importantly, greater weight loss at 12 months was related to higher adherence trajectories.

Consistent with Stecher’s systematic review, the SMARTER study employed a hybrid design that integrated both pragmatic and explanatory components [17]. Pragmatic components included the use of commercial wearables and prescription of MVPA goals in free-living conditions, allowing real-world applicability. However, explanatory elements involved research-specific infrastructure (e.g., ADAM data system) and tailored FB messages delivered by the SMARTER app to facilitate intervention fidelity. Stecher et al. noted that more pragmatic components often demonstrate smaller incremental effects, underscoring the trade-off between internal and external validity [17]. Based on the findings of the SMARTER study, we recommend that future research employ adaptive pragmatic designs that personalize feedback messages to be more responsive to participants’ progressive behavioral changes, or absence of change. Our findings indicate that feedback delivered through a fixed-rule algorithm, without adjustment for changes in participants’ behavior over the course of the intervention, does not improve adherence sufficiently.

Our findings align with prior work by Imes et al. [19] and Adachi et al. [20], who identified four and three step-count trajectories, respectively. Despite differences in intervention modality (in-person vs. remote) and devices (pedometers vs. Fitbits), all studies demonstrated similar MVPA trajectories: trajectory group memberships with high, moderate, and low activity. Similarly to Imes et al., we found that trajectory membership was related to greater weight loss. Although predictors varied, Imes et al. reported no demographic associations, whereas Adachi et al. found that older age and greater app engagement predicted the most active group. These findings suggest that PA trajectories are similar across intervention modalities, but predictors of trajectory group membership are different. Unlike prior step-based interventions [19,20], our study examined adherence to ≥300 min/week of MVPA using Fitbit trackers in a fully remote program with tailored feedback, providing important insights into the potential role of commercial wearables in promoting adherence to MVPA goals without in-person coaching or structured programs.

Our study found that higher first-week MVPA predicted long-term adherence. This finding is consistent with prior research [25,36]. Unick et al. demonstrated that early PA adoption predicts long-term activity among previously sedentary individuals [25], while Stansbury et al. showed that early goal attainment patterns in online obesity treatment were associated with long-term adherence and weight loss [36]. Importantly, SMARTER was fully remote and provided self-monitoring tools to all participants, supplemented by personalized feedback in the SM + FB arm that did not exert a differential effect. This suggests early MVPA may reflect intrinsic motivation or readiness to change. Because early performance monitoring is critical for long-term adherence, future research should assess baseline PA during the pre-trial period using both self-report questionnaires and objective measures. These pre-intervention data would inform an adaptive intervention to target less than adequate PA among non-adherers in the early phase, or reinforcement of high adherence to promote sustained higher levels of PA and improved outcomes.

Our results suggest that older adults and men may respond more positively to wearable trackers. A review of psychosocial factors affecting PA found that women consistently engage in less PA than men, increasing their risk of chronic disease. This disparity is linked to lower self-efficacy, less perceived social support, and motivational factors, such as health, weight management, and physical appearance [37]. Similarly, the Diabetes Prevention Program (DPP) found women, young adults, and non-white participants experienced barriers to weight loss and PA, including self-monitoring problems, social cues, holidays, time management, and low motivation [22]. Practical strategies, such as problem-solving, goal setting, and regular self-monitoring, were effective in helping women and young adults to overcome barriers to PA and improve long-term adherence in the DPP [22]. For groups with lower adherence, mHealth interventions could incorporate flexible scheduling with remotely delivered exercise sessions to overcome distance- and time-related barriers and personalized coaching to enhance self-efficacy [22] and address psychosocial barriers [37].

Although our sample was predominantly female, obesity prevalence in the US is comparable between genders [2]. However, major behavioral interventions, such as DPP and Look AHEAD, have enrolled mostly women (~70%) [22,38], limiting generalizability. More recent studies are examining strategies to address gender differences. Males may perceive weight loss as less relevant to their identity, reducing participation unless recruitment messages emphasize attributes like strength or physical performance rather than general health [39]. A 6-month study of men with overweight or obesity found that a brief newspaper advertisement campaign was the most effective recruitment method, and that, rather than lack of interest, the identified recruitment strategy was the main barrier to male participation in weight loss interventions [40].

Another study of adults with obesity found that targeted recruitment strategies, such as radio ads, direct mail, and in-clinic ads, increased enrollment from 14% to 50% among men and from 13% to 47% among non-white individuals. These studies demonstrate the effectiveness of targeted approaches in improving the participation of males who have been historically underrepresented in weight-loss trials [39,40].

Finally, our findings suggest that there was a significant graded association between MVPA goal adherence and weight loss at 12 months. Although the high-adherence group showed the greatest weight change at 12 months (6.2%), smaller reductions may still result in health benefits [41]. A systematic review of 70 trials found that losing less than 5% of body weight, though below the conventional clinical threshold, was associated with improvements in 60% of 137 assessed health markers, including metabolic, cardiovascular, inflammatory, and quality of life measures, suggesting that 94% of SMARTER participants in lower adherence groups achieved some improvement in risk reduction with a modest weight loss [41].

## 5. Strengths and Limitations

This study’s strengths include a large sample, high retention, and a randomized controlled trial design. It is among the few long-term studies that identify MVPA goal-adherence trajectories, rather than step-based goals [19,20], using commercial wearable activity trackers. This study addressed gaps in research by linking MVPA goal-adherence trajectories to weight loss and offering insights into long-term behavior change for PA in adults with overweight or obesity. The findings highlight the importance of tailoring MVPA goals to baseline characteristics and using early indicators to guide intervention support strategies.

This study has several limitations. First, first-week MVPA levels were used as a proxy for baseline PA, which may overestimate habitual activity due to increased awareness of monitoring (i.e., Hawthorne effect). Obtaining objective measures of usual PA without introducing the Hawthorne effect is challenging, as participants often modify their routines when being monitored. However, introducing a run-in period in which participants wore the device without the prescription of MVPA goals before data collection may have minimized this limitation. Therefore, using the first week of MVPA as baseline PA may have introduced bias, potentially affecting the validity of the obtained results. Self-report questionnaires of PA offer an alternative but are susceptible to recall bias. Future research needs to validate wearable-derived PA measures against research-grade accelerometers and self-report questionnaires to improve the validity of the results. It is commonly accepted that at least 4 wear days are needed to accurately predict habitual PA week for adults [42]. However, extrapolating from four valid wear days to a full week may introduce potential bias if non-wear days, weekends, or low-activity days are included. To address this potential bias, we conducted a sensitivity analysis using only valid days without extrapolation to 7 days, and the results were consistent with the primary analysis (see Supplemental File S1). Nevertheless, future research should consider longer days to minimize potential bias.

Second, the sample was predominantly female and White, which limits the generalizability of the findings to more diverse populations. Although our sample was relatively homogeneous, our findings may have implications for diverse populations. For example, initial MVPA levels could inform adaptive interventions across demographic groups, including non-White participants, males, and younger adults, to tailor interventions according to an individual’s early performance. Finally, the high-adherence trajectory group was relatively small (n = 32, 6.4%), and findings for this group should be interpreted with caution due to limited precision.

Additionally, remote weigh-ins during COVID-19 and use of Fitbit’s “fairly active” and “very active” minutes as proxies for moderate- and vigorous-intensity PA may have introduced measurement errors. Moreover, the intervention’s emphasis on walking may reduce generalizability to higher-intensity PA programs and younger adults performing high-intensity activities. Prior research comparing the Fitbit Charge 2 with the ActiGraph GT3X (Pensacola, FL, USA) found moderate underestimation for light activity and greater underestimation for higher intensities [43]. In a sample of middle-aged adults with overweight or obesity, the Fitbit Charge 2 underestimated MVPA by an average of 31 min/day (95% CI of agreement: −132 to 71 min/day), corresponding to a 34% lower estimate compared to the hip-worn Actigraph GT3X (mean MVPA: 91 vs. 59 min/day [43]. Although ActiGraph devices are considered better alternatives to commercial wearables in terms of their accuracy, their complexity and limited user-friendliness make them impractical for long-term behavioral interventions, whereas commercial wearables like Fitbit are better suited for free-living conditions and sustained engagement. Because walking was the most recommended activity among middle-aged and older adults with overweight or obesity, the potential impact of this measurement error is likely moderate rather than substantial. Furthermore, because all participants received non-personalized Fitbit feedback, this likely acted as a confounder and may have reduced the impact of tailored feedback in the SM + FB group. Finally, technical issues (e.g., firmware updates, sensor errors) may have contributed to misclassification. Generally, activity classification was based on Fitbit’s MET-based categories [31], which align with PA research standards.

## 6. Conclusions

We identified four distinct adherence trajectories to the study-prescribed MVPA goals over 12 months using a commercial wearable activity tracker in an mHealth weight loss trial. Higher adherence was related to greater weight loss, and early MVPA levels predicted long-term adherence. Future research needs to scale the interventions to provide treatment to diverse underserved populations that are burdened with obesity and lack access to behavioral or lifestyle treatment programs. Moreover, future studies need to focus on validating wearable-derived PA measures against research-grade accelerometers and self-report questionnaires and addressing device-related measurement errors. Our findings highlight the need to monitor early adherence to identify individuals at risk of not meeting behavioral goals and to provide timely support during critical periods. Future research needs to build on these findings to develop adaptive interventions that respond dynamically to participants’ responses to interventions.

## Figures and Tables

**Table 1:** Characteristics of SMARTER study participants (N = 502).

Characteristic	Descriptive Statistics
Age, years; mean ± SD	45.0 ± 14.4
Female; n (%)	399 (79.5)
White; n (%)	414 (82.5)
Married/partnered; n (%)	329 (65.5)
Follow-up during the COVID-19; n (%)	262 (52.2)
BMI, kg/m^2^; mean ± SD	33.7 ± 4.0
12-mo PA messages opened for SM + FB, %; mean ± SD	67.0 ± 32.5
First week MVPA; minutes	
mean ± SD	244.1 ± 171.9
median [Q1, Q3]	214 [118, 346]
Met ≥300 min/week during the first week; n (%)	161 (32.1)

Notes: BMI = Body Mass Index; MVPA = Moderate-to-Vigorous-Intensity Physical Activity; PA = Physical Activity; Q1 = 25th Quartile; Q3 = 75th Quartile; SD = Standard Deviation; SM + FB = Self-Monitoring and Feedback Group. The non-white race category included individuals who self-reported as Black (n = 48, 9.6%), Asian (n = 14, 2.8%), or multi-racial (n = 26, 5.2%). Mean ± SD values were reported for normally distributed variables, and median [Q1, Q3] was reported for variables with skewed distributions.

**Table 2:** Percent study-prescribed MVPA goal adherence over 12 months from the final group-based trajectory modeling.

#	Trajectory Group Membership	Polynomial Order	Estimate ± SE	Estimated π 95% CI	*t*-Statistic	*p*-Value
1	Lower stable adherence, n = 173 (34.5%)	Intercept	32.20 ± 0.73	0.34 0.30, 0.39	44.33	<0.0001
2	Moderate adherence, n = 200 (39.8%)	Intercept	82.18 ± 1.87	0.40 0.36, 0.44	44.02	<0.0001
		Linear	−1.29 ± 0.31		−4.23	<0.0001
		Quadratic	0.06 ± 0.01		4.51	<0.0001
		Cubic	−0.0008 ± 0.0002		−4.60	<0.0001
3	Increasing adherence, n = 97 (19.3%)	Intercept	112.38 ± 2.51	0.19 0.16, 0.23	44.78	<0.0001
		Linear	2.69 ± 0.41		6.48	<0.0001
		Quadratic	−0.08 ± 0.02		−4.26	<0.0001
		Cubic	0.0006 ± 0.0002		2.73	0.006
4	High adherence, n = 32 (6.4%)	Intercept	182.44 ± 3.10	0.06 0.04, 0.09	58.76	<0.0001
		Linear	2.14 ± 0.28		7.78	<0.0001
		Quadratic	−0.03 ± 0.01		−6.48	<0.0001
	Sigma		40.01 ± 0.20		198.87	<0.0001

Notes: AIC = Akaike’s Information Criterion; BIC = Bayesian Information Criterion; CI = Confidence Interval; MVPA = Moderate-to-Vigorous-Intensity Physical Activity; SE = Standard Error. π = Estimated group membership probability estimated based on the maximum posterior assignment rule. The final GBTM model statistics were BIC_1_: −103,788.6 (n _repeated assessments_ = 20 669), BIC_2_: −103,725.4 (N = 502), AIC: −103,653.7, log-likelihood: −103,619.7.

**Table 3:** Associations between study-prescribed MVPA goal-adherence trajectories and predictors from the final group-based trajectory modeling.

#	Trajectory Group Membership	Predictors	Estimate ± SE	AOR (95% CI)	*t*-Value	*p*-Value	FDR Correction
1	Lower stable adherence, n = 173 (34.5%)	Reference	---	---	---	---	---
2	Moderate adherence, n =200 (39.8%)	Constant	−4.37 ± 0.83	---	−5.26	<0.0001	---
		Age, years	0.03 ± 0.01	1.03 (1.01–1.05)	2.51	0.01	0.03
		Female vs. Male (reference)	−0.83 ± 0.46	0.44 (0.18–1.07)	−1.83	0.07	0.10
		Partnered vs. Single (reference)	−0.57 ± 0.31	0.57 (0.31–1.04)	−1.85	0.06	0.10
		First-week MVPA, minutes (square-root transformed)	0.45 ± 0.05	1.57 (1.42–1.73)	8.79	<0.0001	<0.0001
		Follow-up during COVID-19: Yes vs. No (reference)	−0.22 ± 0.28	0.80 (0.46–1.39)	−0.81	0.42	0.50
		SM + FB vs. SM (reference)	0.18 ± 0.28	1.20 (0.69–2.07)	0.64	0.52	0.57
3	Increasing adherence, n = 97 (19.3%)	Constant	−8.10 ± 1.10	---	−7.35	<0.0001	---
		Age, years	0.04 ± 0.01	1.04 (1.02–1.06)	3.32	0.0009	0.004
		Female vs. Male (reference)	−1.37 ± 0.51	0.25 (0.09–0.69)	−2.69	0.007	0.02
		Partnered vs. Single (reference)	−1.11 ± 0.38	0.33 (0.16–0.69)	−2.95	0.003	0.01
		First-week MVPA, minutes (square-root transformed)	0.67 ± 0.07	1.95 (1.70–2.24)	10.24	<0.0001	<0.0001
		Follow-up during COVID-19: Yes vs. No (reference)	−0.72 ± 0.35	0.49 (0.25–0.97)	−2.07	0.04	0.07
		SM + FB vs. SM (reference)	0.41 ± 0.35	1.51 (0.76–2.99)	1.17	0.24	0.31
4	High adherence, n = 32 (6.4%)	Constant	−19.23 ± 2.30	---	−8.36	<0.0001	---
		Age, years	0.05 ± 0.02	1.05 (1.01–1.09)	2.46	0.01	0.03
		Female vs. Male (reference)	−1.95 ± 0.70	0.14 (0.04–0.56)	−2.78	0.005	0.02
		Partnered vs. Single (reference)	−0.39 ± 0.64	0.68 (0.19–2.37)	−0.61	0.54	0.57
		First-week MVPA, minutes (square-root transformed)	1.22 ± 0.12	3.39 (2.68–4.29)	9.92	<0.0001	<0.0001
		Follow-up during COVID-19: Yes vs. No (reference)	−0.70 ± 0.58	0.50 (0.16–1.55)	−1.20	0.23	0.31
		SM + FB vs. SM (reference)	−0.01 ± 0.59	1.00 (0.31, 3.23)	−0.01	0.99	0.99

Notes: CI = Confidence Interval; MVPA = Moderate-to-Vigorous-Intensity Physical Activity; AOR = Adjusted Odds Ratio; SE = Standard Error; SM = Self-Monitoring Group; SM + FB = Self-Monitoring plus Feedback Group. In the final model, *p*-values were adjusted using the False Discovery Rate (FDR) correction to account for multiple testing.

**Table 4:** Percent weight change by MVPA goal-adherence trajectory group membership 12 months.

Full Sample (N)/ Completers Only (n)	Percent Weight Change (Full Sample, N = 502) Mean (95% CI)	*p*-Value	Cohen’s d	Percent Weight Change (Completers, n = 394) Mean (95% CI)	*p*-Value	Cohen’s d
Lower stable adherence n = 173/121	−0.33 −1.41, 0.74	0.55	reference	−1.87 −3.12, −0.62	0.003	reference
Moderate adherence n = 200/157	−2.59 −3.58, −1.59	<0.0001	0.32	−3.59 −4.68, −2.49	<0.0001	0.26
Increasing adherence n = 97/87	−4.29 −5.73, −2.86	<0.0001	0.55	−4.77 −6.24, −3.30	<0.0001	0.44
High adherence n = 32/29	−6.16 −8.66, −3.66	<0.0001	0.71	−6.82 −9.37, −4.27	<0.0001	0.75

Notes: CI = Confidence Interval; MVPA = Moderate-to-Vigorous-Intensity Physical Activity.

## Data Availability

The original contributions presented in this study are included in the article/Supplementary Materials. Further inquiries can be directed to the corresponding author.

## Supplementary Materials

The following supporting information can be downloaded at: https://www.mdpi.com/article/10.3390/s25247595/s1, Table S1. Determining the number of trajectory groups. Table S2. Determining the highest-order term of 4 trajectory groups. Table S3. Model fit statistics for the final group-based trajectory model without risk factors. Figure S1. Estimated trajectories of percentage adherence to the study-prescribed MVPA goals with 95% confidence bands over 12 months from group-based trajectory modeling without risk factors. Table S4. Determining the number of trajectory groups. Table S5. Determining the highest-order term of 4 trajectory groups. Table S6. Model fit statistics for the final group-based trajectory model. Figure S2. Estimated trajectories of average percentage adherence to the study-prescribed MVPA goals with 95% confidence bands over 12 months from group-based trajectory modeling without risk factors (recomputed without multiplication by 7 days for sensitivity analysis). Table S7. Mean (95% CI) predicted values for adherence to MVPA goals at weeks 1, 26, and 52 from group-based trajectory modeling without risk-factors. Table S8. Characteristics of SMARTER participants by completion status at 12 months.
