# Supplementary material for: Trajectories of Adherence to Study-Prescribed Physical Activity Goals in a mHealth Weight Loss Intervention
Source: Sensors (Basel). 2025 Dec 15;25(24):7595. doi: 10.3390/s25247595 (PMC12736852; doi:10.3390/s25247595)
Supplement: Supplementary file 1 [file sensors-25-07595-s001.zip › Supplemental S1.pdf]

Supplemental File S1. Group-based trajectory model building process for MVPA goal trajectories

Table S1. Determining the number of trajectory groups

**Number of groups**	**BIC_1(N=20669)**	**BIC_2 (N=502)**	**AIC**	**Smallest group size (%)**
2	-107 145.0	-107 019.0	-106 989.5	25.5
3	-105 209.2	-105 170.1	-105 125.8	16.1
4	-103 942.7	-103 890.7	-103 831.6	6.4
5	-103 518.9	-103 453.8	-103 380.0	5.8
The four-group trajectory model was selected as the optimal solution because it provided the best balance of model fit, parsimony, and adequate group size.				

Table S2. Determining the highest-order term of 4 trajectory groups.

**Function**	**1st iteration**	**2nd iteration**	**3rd iteration**	**4th iteration**	**5th iteration**	**6th iteration**
Group 1	quintic (*p*-value=0.10)	quartic (*p*-value =0.05)	cubic (*p*-value =0.06)	quadratic (*p*-value =0.39)	linear (*p*-value =0.07)	intercept *p*-value <0.0001)
Group 2	quintic (*p*-value=0.06)	quartic (*p*-value =0.32)	cubic (*p*-value <0.0001)	cubic (*p*-value <0.0001)	cubic (*p*-value <0.0001)	cubic (*p*-value <0.0001)
Group 3	quintic (*p*-value =0.46)	quartic (*p*-value =0.82)	cubic (*p*-value =0.006)	cubic (*p*-value =0.006)	cubic (*p*-value =0.006)	cubic (*p*-value =0.006)
Group 4	quintic (*p*-value =0.10)	quartic (*p*-value =0.14)	cubic (*p*-value =.11)	quadratic (*p*-value <0.0001)	quadratic (*p*-value <.0001)	quadratic (*p*-value <0.0001)
Model fit						
BIC_1 (N=20,669)	-103,942.7	-103,927.7	-103,911.4	-103, 904.6	-103,900.0	-103,896.7
BIC_2 (N=502)	-103,890.7	-103,883.1	-103,874.2	-103, 871.1	-103,868.4	-103,867.0
AIC	-103 831.6	-103,832.5	-103,832.0	-103, 833.1	-103,832.5	-103,833.2

Notes: AIC = Akaike's Information Criterion; BIC = Bayesian Information Criterion; MVPA = Moderate-to-Vigorous-Intensity Physical Activity. Percentage of adherence to MVPA goals was computed with the following formula: $\frac{\text{(fairly active+very active minutes) x 7 days}}{\text{number of days meeting} \geq 500 \text{ daily steps for} \geq 4 \text{ days/week}}$ x 100%.

Table S3. Model fit statistics for the final group-based trajectory model without risk factors

**Group**	**Prevalence (%) (N=502)**	**Average group posterior probability (> 70%)**	**Odds of correct classification (> 5.0)**
Group 1	34.5	97.1	63.44
Group 2	40.2	97.1	49.80
Group 3	18.9	98.7	325.70
Group 4	6.4	99.8	7298.00

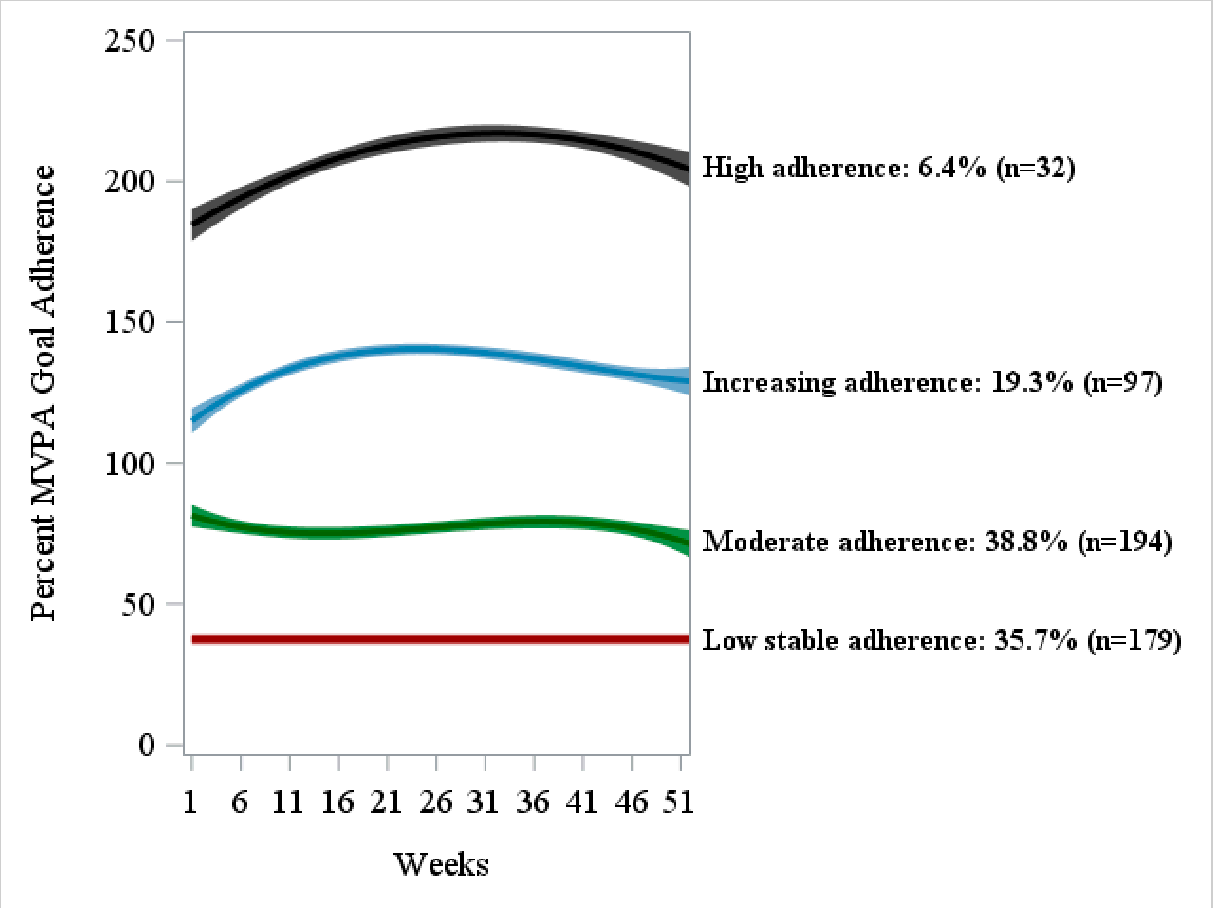

Figure S1. Estimated trajectories of percentage adherence to the study-prescribed MVPA goals with 95% confidence bands over 12 months from group-based trajectory modeling without risk factors

Notes: MVPA = Moderate- to-Vigorous-Intensity Physical Activity. Percentage of adherence to MVPA goals was computed with the following formula: $\frac{\text{(fairly active+very active minutes) x 7 days}}{\text{number of days meeting} \geq 500 \text{ daily steps for} \geq 4 \text{ days/week}}$ over ≥300 minutes/week x 100%.

Group-based trajectory model building process for average MVPA goal trajectories (recomputed without multiplication by 7 days)

Table S4. Determining the number of trajectory groups.

Number of groups	BIC_1(N=20669)	BIC_2 (N=502)	AIC	Smallest group size (%)
2	-67 955.60	-67 929.58	-67 900.05	25.5
3	-66 119.73	-66 080.69	-66 036.40	16.1
4	-64 853.29	-64 801.24	-64 742.18	6.4
5	-64 429.41	-64 364.35	-64 290.52	5.8
The four-group trajectory model was selected as the optimal solution because it provided the best balance of model fit, parsimony, and adequate group size.				

Table S5. Determining the highest-order term of 4 trajectory groups.

Function	1st iteration	2nd iteration	3rd iteration	4th iteration	5th iteration	6th iteration
Group 1	quintic (*p*-value=0.11)	quartic (*p*-value =0.05)	cubic (*p*-value =0.06)	quadratic (*p*-value =0.39)	linear (*p*-value =0.07)	intercept *p*-value <0.0001)
Group 2	quintic (*p*-value=0.06)	quartic (*p*-value =0.32)	cubic (*p*-value <0.0001)	cubic (*p*-value <0.0001)	cubic (*p*-value <0.0001)	cubic (*p*-value <0.0001)
Group 3	quintic (*p*-value =0.51)	quartic (*p*-value =0.82)	cubic (*p*-value =0.006)	cubic (*p*-value =0.006)	cubic (*p*-value =0.006)	cubic (*p*-value =0.006)
Group 4	quintic (*p*-value =0.11)	quartic (*p*-value =0.14)	cubic (*p*-value =0.11)	quadratic (*p*-value <0.0001)	quadratic (*p*-value <0.0001)	quadratic (*p*-value <0.0001)
Model fit						
BIC_1 (N=20669)	-64,853.29	-64,838.25	-64,821.96	-64,815.13	-64,810.54	-64,807.26
BIC_2 (N=502)	-64,801.24	-64,793.63	-64,784.79	-64,781.67	-64,778.94	-64,777.52
AIC	-64,742.18	-64,743.01	-64,742.60	-64,743.71	-64,743.08	-64,743.77

Notes: AIC = Akaike's Information Criterion; BIC = Bayesian Information Criterion; MVPA = Moderate-to-Vigorous-Intensity Physical Activity. Average percentage of adherence to MVPA goals was computed with the following formula: $\frac{\text{fairly active+very active minutes}}{\text{number of days meeting} \geq 500 \text{ daily steps for} \geq 4 \text{ days/week}}$ over ≥300 minutes/week x 100%.

Table S6. Model fit statistics for the final group-based trajectory model.

Group	Prevalence (%) (N=502)	Average group posterior probability (> 70%)	Odds of correct classification (> 5.0)
Group 1	n = 170, 35.7%	97.1	60.34
Group 2	n= 202, 38.7%	97.1	53.05
Group 3	n = 95, 19.3%	98.7	317.70
Group 4	n = 32, 6.4%	99.8	7299.00

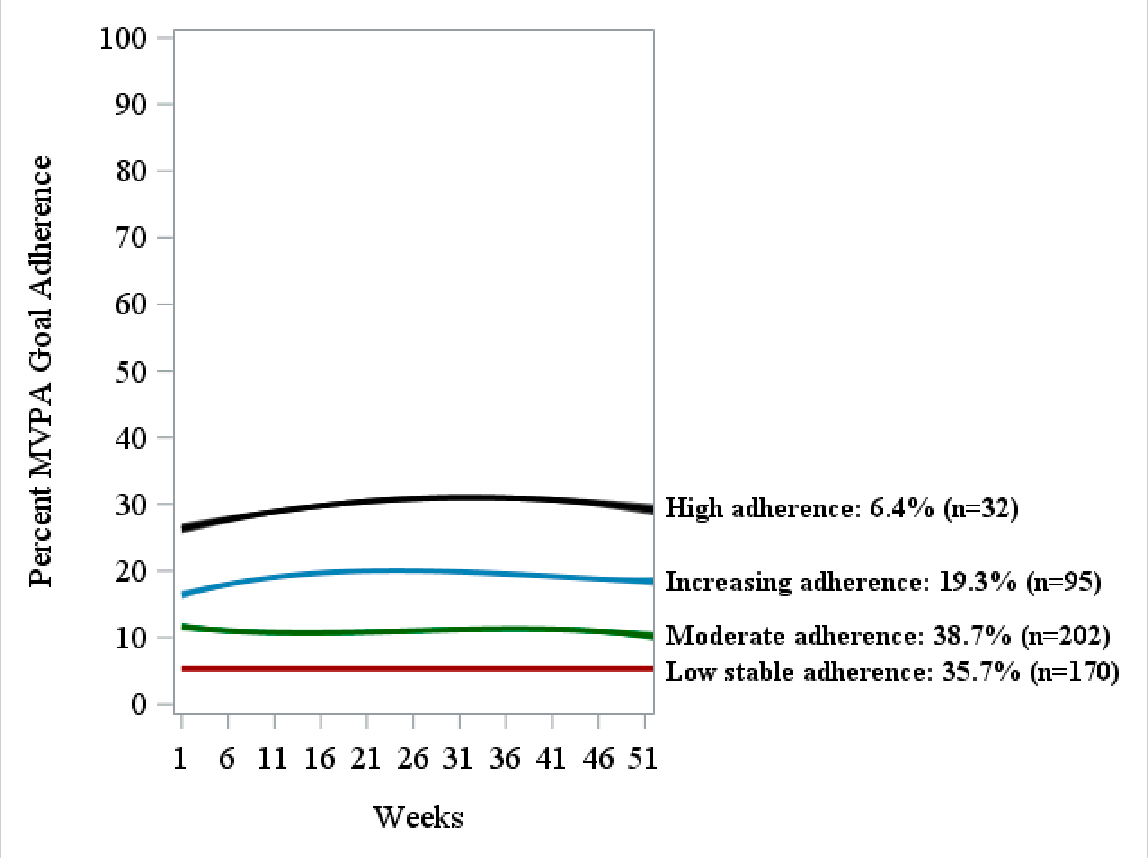

Figure S2. Estimated trajectories of average percentage adherence to the study-prescribed MVPA goals with 95% confidence bands over 12 months from group-based trajectory modeling without risk factors (recomputed without multiplication by 7 days for sensitivity analysis).

Notes: MVPA = Moderate- to-Vigorous-Intensity Physical Activity. Percentage of adherence to MVPA goals was computed with the following formula: $\frac{\text{fairly active+very active minutes}}{\text{number of days meeting} \geq 500 \text{ daily steps for} \geq 4 \text{ days/week}}$ over ≥300 minutes/week x 100%.

Table S7. Mean (95% CI) predicted values for adherence to MVPA goals at weeks 1, 26, and 52 from group-based trajectory modeling without risk-factors.

	**Percentage of Adherence to MVPA goals**				**Average Percentage of Adherence to MVPA goals** *(recomputed without multiplication by 7 days)*			
	Low stable adherence	Moderate adherence	Increasing adherence	High adherence	Low stable adherence	Moderate adherence	Increasing adherence	High adherence
Week 1	37.49<br>35.61, 39.37	81.33<br>77.46, 85.20	115.00<br>110.58, 119.41	184.58<br>178.93, 190.24	5.36<br>5.09, 5.62	11.62<br>11.07, 12.17	16.43<br>15.80, 17.06	26.37<br>25.56, 27.18
Week 26	37.49<br>35.61, 39.37	77.22<br>75.24, 79.20	140.38<br>138.55, 142.20	215.74<br>212.64, 218.85	5.36<br>5.09, 5.62	11.03<br>10.75, 11.31	20.05<br>19.79, 20.31	30.82<br>30.38, 31.26
Week 52	37.49<br>35.61, 39.37	71.31<br>66.45, 76.17	129.01<br>124.02, 134.00	204.12<br>198.00, 210.24	5.36<br>5.09, 5.62	10.19<br>9.49, 10.88	18.43<br>17.72, 19.14	29.16<br>28.29, 30.03

Notes: CI = Confidence Interval; MVPA = Moderate- to-Vigorous-Intensity Physical Activity. Originally, percentage of adherence to MVPA goals was computed with the following formula: $\frac{\text{(fairly active+very active minutes) x 7 days}}{\text{number of days meeting ≥500 daily steps for ≥4 days/week}}$ over ≥300 minutes/week x 100%. For sensitivity analysis, average percentage adherence to MVPA goals was recomputed without multiplication by 7 days: $\frac{\text{fairly active+very active minutes}}{\text{number of days meeting ≥500 daily steps for ≥4 days/week}}$ over ≥300 minutes/week x 100%.
