# Supplementary material for: Trajectories of Adherence to Study-Prescribed Physical Activity Goals in a mHealth Weight Loss Intervention
Source: Sensors (Basel). 2025 Dec 15;25(24):7595. doi: 10.3390/s25247595 (PMC12736852; doi:10.3390/s25247595)
Supplement: Supplementary file 1 [file sensors-25-07595-s001.zip › Supplemental S2.pdf]

Supplemental File S2

Table S8. Characteristics of SMARTER participants by completion status at 12 months.

	**Total (N=502)**	**Completed (n=394)**	**Dropout (n=108)**	**p-value**
SM+FB; n (%)	251 (50%)	202 (51.3%)	49 (45.4%)	0.28
Age, years; mean ± SD	45.0 ± 14.4	47.4 ± 14.2	36.1 ± 11.5	<0.0001
Female; n (%)	399 (79.5%)	310 (78.7%)	89 (82.4%)	0.40
White; n (%)	423 (84.3%)	337 (85.5%)	86 (79.6%)	0.14
Married/partnered; n (%)	329 (65.5%)	264 (67%)	65 (60.2%)	0.19
BMI, kg/m²; mean ± SD	33.7 ± 4.0	33.5 ± 3.8	34.6 ± 4.3	0.001
Follow-up during the COVID-19; n (%)	264 (52.6%)	200 (50.8%)	64 (59.3%)	0.11
First-week MVPA, minutes				
mean ± SD	245.5 ± 171.4	255.6 ± 173.8	208.8 ± 157.6	0.01
median [Q1, Q3]	215 (119, 346)	218 [126, 354]	176 [80, 305]	0.01

Notes: BMI = Body Mass Index; MVPA = Moderate- to-Vigorous-Intensity Physical Activity; PA = Physical Activity; Q1 = 25th Quartile; Q3 = 75th Quartile; SD = Standard Deviation; SM+FB = Self-Monitoring and Feedback Group. The non-white race category included individuals who self-reported as Black (n = 48, 9.6%), Asian (n = 14, 2.8%), or multi-racial (n = 26, 5.2%). Mean ± SD values were reported for normally distributed variables, and median [Q1, Q3] was reported for variables with skewed distributions.
